# Early Exercise Rehabilitation Enhances Gait Following Endoprosthetic Knee Reconstruction for Bone Tumors: A Systematic Review and Meta-Analysis

**DOI:** 10.7759/cureus.91384

**Published:** 2025-09-01

**Authors:** Yi-Hong Wu

**Affiliations:** 1 Department of Physical Medicine and Rehabilitation, Cathay General Hospital, Taipei, TWN

**Keywords:** endoprosthesis, exercise, gait, knee reconstruction, timed up and go

## Abstract

Endoprosthetic knee replacement after bone tumor resection is a critical limb-salvage procedure, yet patients often experience persistent gait abnormalities that hinder recovery and quality of life. Few studies have focused on the effects of exercise training on gait and functional ambulation, and potential time-response relationships between exercise training and outcomes remain unclear. We aimed to conduct a systematic review and meta-analysis to evaluate the effect of exercise training on gait. We also aimed to propose a rehabilitation protocol based on state-of-the-art evidence.

A systematic search was conducted on databases including MEDLINE, Web of Science, Google Scholar, Cochrane, and Embase following Preferred Reporting Items for Systematic reviews and Meta-Analyses (PRISMA) guidelines. Studies assessing gait following exercise training in patients after endoprosthetic knee reconstruction were included. Three controlled clinical studies with 576 patients met the inclusion criteria. The pooled effect size was moderate, with Hedges’ g for TUG (Timed Up and Go) being -0.569 (95% CI: -0.597 to -0.540). More complex gait parameters showed modest, nonsignificant improvements, particularly when rehabilitation was initiated later.

Early structured rehabilitation meaningfully enhances gait symmetry, walking speed, and balance control in patients undergoing knee endoprosthetic replacement after tumor resection. The integration of timely, individualized exercise programs into standard postoperative care may maximize functional recovery and improve mobility.

## Introduction and background

Endoprosthetic knee reconstruction following bone tumor resection is a limb-salvage procedure for resectable bone tumors, including low-grade bony sarcomas, recurrent aggressive benign tumors, and carefully selected high-grade sarcomas with large tumor defects. It is designed to restore joint stability and preserve mobility. While the procedure is highly effective in maintaining structural integrity and reducing the need for amputation, many patients experience persistent gait abnormalities. These include Trendelenburg gait, abnormal hip positioning, knee hyperextension or sustained flexion, and atypical ankle movements such as excessive plantarflexion or dorsiflexion [1]. Such gait deviations are particularly common after distal femur or proximal tibia replacements and can significantly hinder postoperative rehabilitation and return to functional independence [2-4]. Furthermore, patients with lower-limb megaprostheses exhibit reduced walking speed, decreased cadence, shorter stride length, and altered joint mechanics compared to healthy controls [4,5]. These gait problems in endoprosthetic knee reconstruction lead to greater energy expenditure than in patients undergoing total knee replacement, as compensation is required for abnormal knee joint conditions during walking and to maintain body balance [6].

The operation site has been shown to affect gait kinematics differently [1,7]. Endoprosthetic reconstruction of the distal femur and proximal tibia presents unique biomechanical challenges (see Table 1). In proximal tibia reconstructions, the knee often presents with a more flexed angle due to significant weakness or disruption of the extensor mechanism, including the quadriceps and patellar tendon [1]. This extensor lag compromises knee extension and leads to poor hip compensatory mechanics, with the hip frequently held in flexion during early stance phases. Additionally, harvesting of the medial gastrocnemius flap results in diminished plantarflexion strength, causing increased ankle dorsiflexion and potential tibial anterior translation during gait. Conversely, distal femur reconstructions generally present with a knee held in extension postoperatively, facilitated by hip extension compensations that load the ground reaction force anteriorly to reduce quadriceps demand during stance. Due to compensatory calf muscle contractions that stabilize the tibia, the ankle demonstrates reduced dorsiflexion [1].

**Table 1:** Comparison of Biomechanical Deficits and Soft Tissue Risks in Proximal Tibia Versus Distal Femur Reconstructions This table summarizes the key kinematic abnormalities, compensatory mechanisms, and soft tissues involved in proximal tibia and distal femur endoprosthetic reconstructions. It highlights differences in knee, hip, and ankle joint positioning, the primary soft tissues involvement in surgery, and the main rehabilitation concerns critical for tailored postoperative management.

Aspect	Proximal Tibia Reconstruction	Distal Femur Reconstruction
Kinematics	Knee: flexed (extension lag from weak/patched extensor mechanism)	Knee: extended (loss of gastrocnemius support)
	Hip: flexed (weaker early stance support);	Hip: extended (ground reaction force forward → less quad demand)
	Ankle: dorsiflexed (gastrocnemius flap → ↓ plantarflexion → tibial anterior translation)	Ankle: reduced dorsiflexion (due to quad weakness, calf contraction compensates stabilizing tibia)
Soft tissues involved in surgery	Patellar tendon to prosthesis	Medial gastrocnemius (flap)
	Medial gastrocnemius (flap)	Vastus medialis (resected)
	Soleus (splitted)	Sartorius (released)
	Tibialis anterior (nerve preserved)	Patellar retinaculum
Main concerns	Long-term extension recovery	Long-term flexion restoration
	Protect extensor mechanism and calf function	

While multimodal rehabilitation strategies, including early mobilization and telerehabilitation, have shown benefits for range of motion, pain control, and subjective functional outcomes [5], the direct impact of structured exercise training on objective gait parameters remains underexplored in this patient population. A previous systematic review of 28 studies aimed to summarize fitness, function, and exercise responses after lower-limb reconstruction with megaprostheses but identified no studies directly evaluating exercise training responses at the time of the review [8]. Since then, few clinical studies have attempted to address this gap, yet no reviews have comprehensively described the effect of exercise training on gait in individuals following limb-salvage surgery with a knee endoprosthesis. In particular, little is known about how such interventions influence spatiotemporal, kinematic, and kinetic measures of gait across different time points post-surgery. Therefore, we conducted a systematic review and meta-analysis to evaluate the effect of exercise training on gait. We also aimed to propose an evidence-based, targeted, structured rehabilitation protocol to minimize functional impairments by specifically addressing the biomechanical and neuromuscular deficits associated with large joint reconstructions.

## Review

Method

This systematic review and meta-analysis focused on controlled clinical trials involving patients with bone tumors who underwent endoprosthetic knee reconstruction, specifically examining the effects of exercise-based rehabilitation on gait-related outcomes. Databases searched included MEDLINE, Web of Science, Google Scholar, Cochrane, and Embase, using terms such as “Gait”, “Exercise”, “Rehabilitation”, “Endoprosthetic”, “Endoprosthesis”, “Prosthetic”, “Prosthesis”, “Limb-Salvage Surgery”, and “Limb-Salvaging Surgery”. Controlled clinical studies reporting quantitative gait parameters following postoperative exercise in distal femur or proximal tibia tumor survivors undergoing limb-salvage surgery were included. Studies were excluded if (1) the bone tumors were not around the knee, (2) patients did not undergo endoprosthetic reconstruction, (3) gait outcomes were not discussed, or (4) no explicit exercise intervention was mentioned. Records written in English up to May 2025 were screened for eligibility by Y.H. Wu and an independent investigator (Yu-Chi Wu). A third investigator (Siang-Ruei Wu) was consulted in cases of disagreement between the two reviewers. The investigators were well informed of the study aim and design but did not contribute to manuscript preparation.

Gait data were extracted, as were other study characteristics, including patient number in either exercise or control group, surgery performed and the endoprosthesis used, exercise training programs, assessment time, and evaluated outcomes. Study quality was assessed using the PEDro scale. This review was not registered in a public database. Zotero was used in the screening process.

For each study, effect sizes were calculated using Hedges’ g, chosen for its bias correction in small samples, to quantify differences in key gait outcomes such as the Timed Up and Go (TUG) test, Timed Up and Down Stairs (TUDS), Gait Deviation Index (GDI), Gait Profile Score (GPS), walking speed, Musculoskeletal Tumor Society Score (MSTS), Toronto Extremity Salvage Score (TESS), and center of mass (CM) velocity during sit-to-stand transitions, a validated measure of balance control.

When only 95% confidence intervals (CI) or interquartile ranges (IQR) were provided in the original studies, we derived standard deviations by back-calculating from the reported statistics with the following formulas. We derived the standard deviation from the IQR with

$SD \approx \frac{\text{IQR}}{1.35}$ 

We calculated the standard deviation from the 95% CI interval via

$SD = SE \cdot \sqrt{n}$

where 

$SE = \frac{CI_{\text{high}} - CI_{\text{low}}}{2 \cdot t_{0.975, df}}$

where

$t_{0.975, df} = \texttt{scipy.stats.t.ppf}(0.975, df)$

where df is the degrees of freedom, which is sample size - 1, with a two-tailed t-distribution and the scipy.stats package of Python being used.

This derivation incorporated the appropriate t-critical values based on the degrees of freedom corresponding to each study’s sample size, ensuring accurate and consistent estimation of variability. This methodological step enhances the precision of effect size calculations and the reliability of subsequent analyses. All statistical analyses were performed using the following packages and languages: scipy version 1.16.0 (SciPy Developers, Austin, Texas) and numpy version 2.0.2 (NumPy Developers, Austin, Texas) in Python 3.7 (Python Software Foundation, Beaverton, Oregon), and meta version 8.2.0 (Guido Schwarzer, Freiburg, Germany) and dplyr version 1.1.4 (RStudio, Boston, Massachusetts) in R (R Foundation for Statistical Computing, Vienna, Austria).

Due to variable exercise program designs and different initiation times of intervention in each included study, a random-effects meta-analysis model was employed to synthesize the pooled effect size for TUG outcomes across studies, accounting for between-study heterogeneity. The Hartung-Knapp adjustment was applied to provide more conservative and robust confidence intervals, which is particularly important given the limited number of included studies. Funnel plots were generated to assess reporting bias, with the standard errors accounting for the amount of heterogeneity estimated by the model. To address the potential influence of individual studies on the overall findings, sensitivity analyses were conducted by sequentially excluding each study and recalculating pooled estimates.

Results

**Figure 1:**
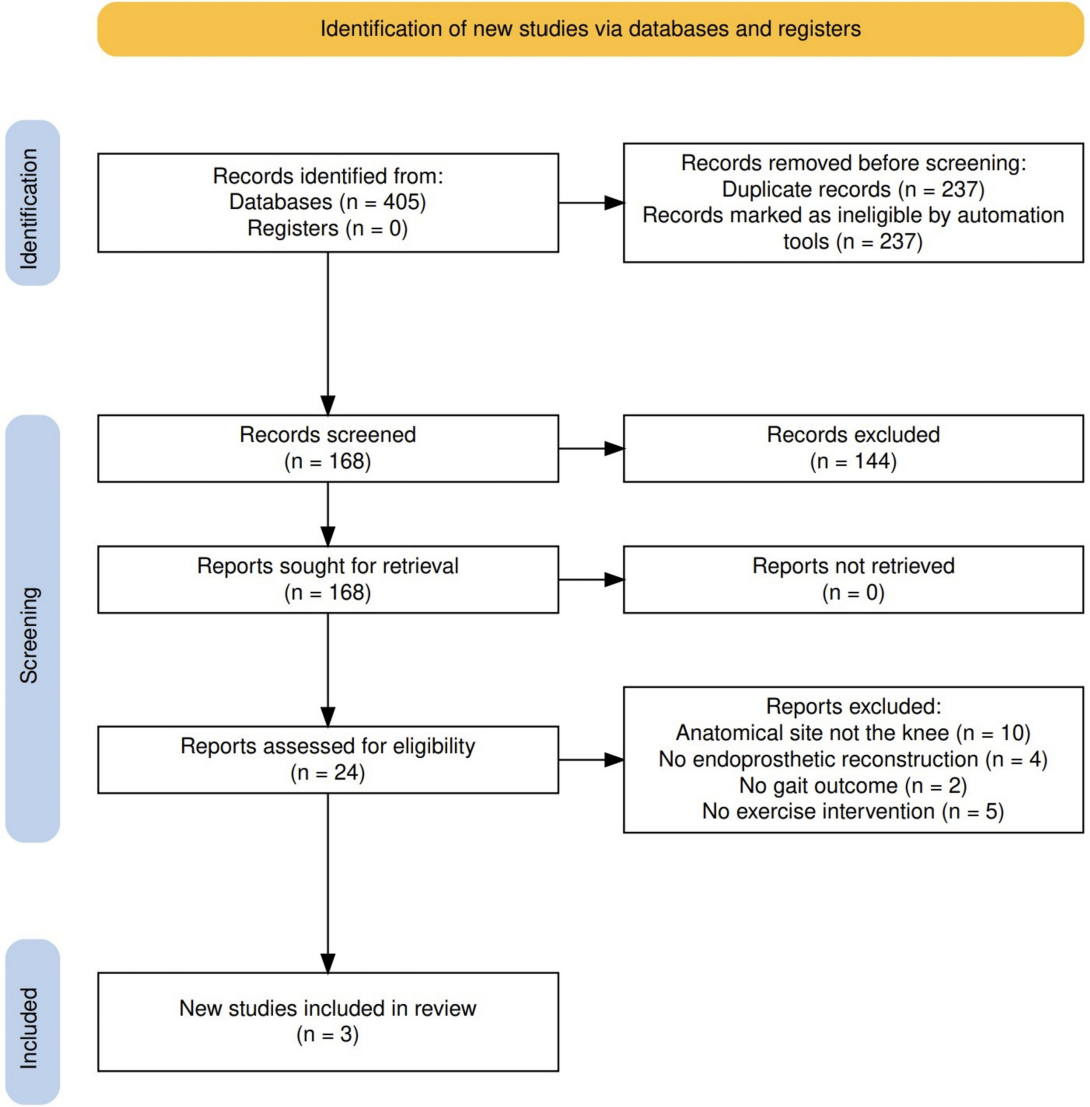
PRISMA 2020 Flow Diagram for Study Selection Flow diagram illustrating the identification, screening, and inclusion of studies in the systematic review on the effects of exercise on gait following endoprosthetic knee reconstruction. A total of 405 records were identified through database searches. After removing 237 duplicates and automatically excluded records, 168 records were screened. Of these, 144 were excluded based on title and abstract. Full-text assessment was conducted on 24 reports, from which 21 were excluded for the following reasons: anatomical site not the knee (n = 10), no endoprosthetic reconstruction (n = 4), no gait outcome (n = 2), and no exercise intervention (n = 5). Three studies were included in the final review. PRISMA: Preferred Reporting Items for Systematic Reviews and Meta-Analyses.

After applying the inclusion and exclusion criteria, three controlled clinical trials involving a total of 576 participants were included in this meta-analysis [9-11]. The inclusion and exclusion process is summarized in Figure 1, and the characteristics of these studies are shown in Tables 2, 3. Two studies assessed gait outcomes 12 months after surgery following 6 months of exercise training [9,11], and one study assessed gait outcomes at least 12 months after surgery after an eight-week exercise program [10]. In two studies, follow-up lasted more than a year [10,11]. One study was randomized with baseline comparability [10], while the others were cohort studies with non-randomized control groups [9,11]. 

**Table 2:** Characteristics of Included Studies on Exercise-Based Rehabilitation Following Endoprosthetic Knee Replacement for Bone Tumors. Summary of key features and outcomes. Interventions varied in intensity, duration, and delivery mode (in-person vs. remote), with consistent evidence favoring structured, progressive exercise over conventional or passive rehabilitation. POD: post-operative day, BAPS: Biomechanical Ankle Platform System, CPM: Continuous Passive Motion, APE: Active Physical Exercise, MET: Metabolic Equivalent of Task, LE: Lower Extremity, GDI: Gait Deviation Index, TESS: Toronto Extremity Salvage Score, MSTS: Musculoskeletal Tumor Society Score, TUG: Timed Up and Go, TUDS: Timed Up and Down Stairs.

Study	Number (Exercise/Control)	Surgery	Control	Intervention	Method	Outcome
Morri et al., 2019 [9]	37 (22/15)	Modular knee prosthesis	Modular knee prosthesis with conventional physiotherapy without balance training	Game-based functional balance training, initiated POD1, 6-month program, 2×/day, 5×/week (450 min/week); strength + proprioception + task-specific exercises using Wii Fit board with visual feedback when partial loading permitted.	Warm-up + stretching, isometric + dynamic strength, sit-to-stand, unilateral/bilateral knee control, stair climbing, backward/lateral walking, cycling, treadmill. Balance program included braiding, tandem walk, foam/BAPS board. Used visual feedback for dynamic weight shifting. Initiated on post-op day 1, progressed over 6 months.	Median walking speed difference: +0.22 m/s (p=0.022); median center of mass speed reduced by 4.5 mm/s (p=0.005). Notable postural control improvement in sit-stand.
Basteck et al., 2022 [10]	11 (6/5)	Megaendoprosthesis around the knee in adolescents/young adults	Booklet with general info on physical activity, no exercise prescription	PROGAIT: Personalized Zoom-supervised 8-week home program, 2×/week. Focus: strength, balance, coordination, LE mobility. Emphasis on extensor/flexor/adductor/abductor muscle strengthening and proprioceptive training for trunk/LE.	Sessions gradually shifted toward independent performance, encouraging self-management. Exercises targeted gait function and postural stability. Interventions performed at least 1 year post-surgery.	Small–medium positive effects on GDI, subjective functional scores TESS and MSTS, and functional tests TUG and TUDS
Du et al., 2024 [11]	528 (264/264)	Endoprosthetic knee reconstruction	Continuous Passive Motion (CPM) for 6 months (same frequency/duration as APE group)	Exercise: 6-month protocol, 5×/week, 2×/day, 40 min/session (400 min/week). Three-stage program by surgical site (tibia/femur), progressively increased MET-based intensity (1.5 → 3.5 METs). Included ROM, strength, balance, stair, and weight-shifting exercises. Personal MET tailored.	Post-op specific structured rehab: Ankle pumps, quad/glute isometrics, passive knee ROM, center-shifting, restricted/then unrestricted ROM, quadriceps strengthening <120°, step training (healthy up, affected down). Specific progression for femur and tibia. Training differentiated by recovery phase (3 stages). Assessments at 3, 6, 12 months. Followed for 5 years.	Statistically significant functional improvement in exercise vs control across all timepoints (3, 6, 12 months). Very large effect sizes at 3 months (Hedges' g > 1.3), moderate effects sustained to 12 months. Quality of life and functional knee recovery significantly better in exercise group. Distal femur patients better than proximal tibia patients in early recovery.

**Table 3:** PEDro Scores of Included Studies on Exercise-Based Rehabilitation After Endoprosthetic Knee Replacement for Bone Tumors. Summary of study quality, sample size, and blinding status for the three included clinical trials.

Variables	Morri et al., 2019 [9]	Basteck et al., 2022 [10]	Du et al., 2024 [11]
Eligibility criteria	Yes	Yes	Yes
Random allocation	No	Yes	No
Concealed allocation	No	Yes	No
Baseline comparability	No	Yes	No
Blinded participants	No	No	No
Blinded therapists	No	No	No
Blinded assessors	No	No	No
Adequate follow-up	No	Yes	Yes
Intention-to-treat analysis	Yes	Yes	Yes
Between-group comparisons	Yes	Yes	Yes
Point estimates and variability	Yes	Yes	Yes
Total PEDro score	4	8	5
Sample size ≥ 50	No	No	Yes

Across all studies, active rehabilitation was associated with significant improvements in postural control, balance, and walking performance. Specifically, balance and exercise training initiated immediately post-surgery led to an increase in walking speed (Hedges’ g = 0.23, 95% CI (0.43, 0.89)) and a reduction in center of mass velocity during sit-to-stand transitions (Hedges’ g = -1.56, 95% CI (-2.31, -0.81)), indicating enhanced balance control. In contrast, interventions initiated one year post-surgery produced small-to-moderate improvements in the Gait Deviation Index (GDI), Gait Profile Score (GPS), and Timed Up and Down Stairs (TUDS), though these changes were not statistically significant (Table 4). Other measures, such as the Musculoskeletal Tumor Society (MSTS) score and the Toronto Extremity Salvage Score (TESS), demonstrated smaller, nonsignificant effects.

**Table 4:** Effects of Exercise-Based Rehabilitation on Gait and Balance Parameters. Mean difference, standard deviations (SD), t-value, degrees of freedom (df), Hedges' g, corresponding 95% confidence intervals (CI), and other test statistics for various gait parameters, including 10-meter walk test (10mWT), center of mass speed, 6-minute walk test (6mWT), Musculoskeletal Tumor Society Score (MSTS), Toronto Extremity Salvage Score (TESS), Gait Deviation Index (GDI), and Gait Profile Score (GPS). The GDI measures overall gait abnormality, while the GPS evaluates the magnitude of deviations in gait patterns. p-values are reported to indicate statistical significance. The p-values < 0.001 were below the reporting threshold and are presented as such.

Measure	Mean difference	SD (Exercise)	SD (Control)	t-value (df)	Welch’s df	p-value (t)	Hedges' g	CI Lower	CI Upper	p-value (g)
10mWT (m/s) [9]	0.22	0.37	0.44	1.58	26.44	0.126	0.53	-0.13	1.20	0.116
Center of mass speed (mm/s) [9]	-4.50	1.85	3.85	-4.21	18.46	< 0.001	-1.56	-2.31	-0.81	< 0.001
6mWT (m) [9]	25.50	35.56	165.93	0.59	14.88	0.567	0.23	-0.43	0.89	0.494
MSTS (%) [9]	-5.00	17.26	12.96	-1.01	34.60	0.322	-0.33	-0.99	0.33	0.354
TESS (%) [9]	-4.00	12.59	9.63	-1.09	34.45	0.282	-0.36	-1.02	0.30	0.191
TUDS (s) [10]	-0.70	3.56	4.11	-0.30	8.05	0.773	-0.17	-1.36	1.02	0.782
GDI (affected) [10]	1.10	14.70	13.65	0.13	8.85	0.901	0.07	-1.01	1.16	0.899
GDI (unaffected) [10]	0.10	17.07	16.94	0.41	8.67	0.693	0.23	-0.86	1.31	0.685
GPS (affected) [10]	4.20	3.12	2.26	0.06	8.88	0.952	0.03	-1.05	1.12	0.952
GPS (unaffected) [10]	-0.30	2.43	2.26	-0.21	8.84	0.837	-0.12	-1.20	0.97	0.834
GPS (overall) [10]	-0.10	2.93	2.39	-0.06	9.00	0.952	-0.03	-1.12	1.05	0.951

Detailed analysis of Timed Up and Go (TUG) test data across studies is provided in Table 5. Individual study results showed variable effect sizes with Morri et al. (2019) reporting a Hedges' g of -0.605 (95% CI (-1.276, 0.066)), Basteck et al. (2022) showing inconclusive results, and Du et al. (2024) demonstrating robust improvements at multiple time points up to five years post-intervention (e.g., Hedges’ g = -0.567 at 12 months) [7-9].

**Table 5:** Effect Sizes (Hedges’ g) and 95% Confidence Intervals for TUG Across Included Studies. Summary of the mean difference, standard deviation (SD), t-value, degrees of freedom (df), calculated Hedges’ g with 95% confidence intervals (CI), and other test statistics for Timed Up and Go (TUG) across included studies, showing group comparisons at various follow-up intervals. The p-values < 0.001 were below the reporting threshold and are presented as such.

Study	Comparison	Mean difference	SD (Exercise)	SD (Control)	t-value (df)	Welch’s df	p-value (t)	Hedges' g	CI Lower	CI Upper	p-value (g)
Morri et al., 2019 [9]	Game vs. Control at 1 Year	-1.40	1.33	3.19	-1.61	17.38	0.126	-0.61	-1.28	0.07	0.077
Basteck et al., 2022 [10]	Exercise vs. Control at 8 Weeks	-0.40	0.64	0.72	-0.97	8.21	0.361	-0.54	-1.75	0.67	0.380
Du et al., 2024 [11]	APE-F vs CPM-F at 3 Months	-1.79	1.27	1.33	-12.65	335.29	< 0.001	-1.37	-1.61	-1.14	< 0.001
	APE-T vs CPM-T at 3 Months	-1.73	1.12	1.04	-13.00	259.18	< 0.001	-1.60	-1.87	-1.32	< 0.001
	APE-F vs CPM-F at 6 Months	-0.99	1.24	1.47	-6.57	313.76	< 0.001	-0.73	-0.95	-0.50	< 0.001
	APE-T vs CPM-T at 6 Months	-1.34	0.90	1.22	-10.05	237.36	< 0.001	-1.25	-1.51	-0.98	< 0.001
	APE-F vs CPM-F at 12 Months	-0.59	1.16	1.01	-4.81	307.21	< 0.001	-0.54	-0.77	-0.32	< 0.001
	APE-T vs CPM-T at 12 Months	-0.53	1.01	0.66	-4.88	209.65	< 0.001	-0.62	-0.88	-0.37	< 0.001
	Pooled APE vs CPM at 3 Months	-1.78	1.72	1.70	-12.80	599.67	< 0.001	-1.04	-1.21	-0.87	< 0.001
	Pooled APE vs CPM at 6 Months	-1.15	1.13	1.43	-10.79	553.02	< 0.001	-0.89	-1.06	-0.72	< 0.001
	Pooled APE vs CPM at 12 Months	-0.57	1.10	0.88	-6.72	532.85	< 0.001	-0.57	-0.74	-0.40	< 0.001

**Figure 2:**
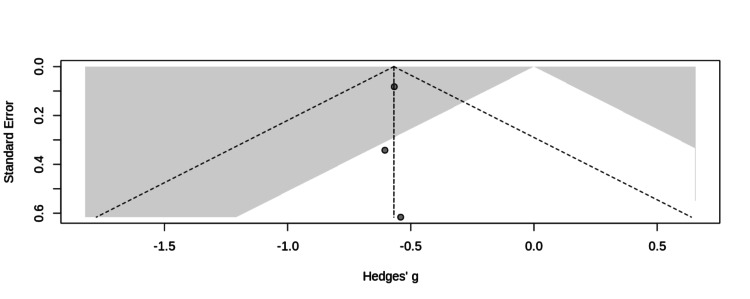
Funnel Plot. p-value < 0.05 in the gray area.

**Figure 3:**
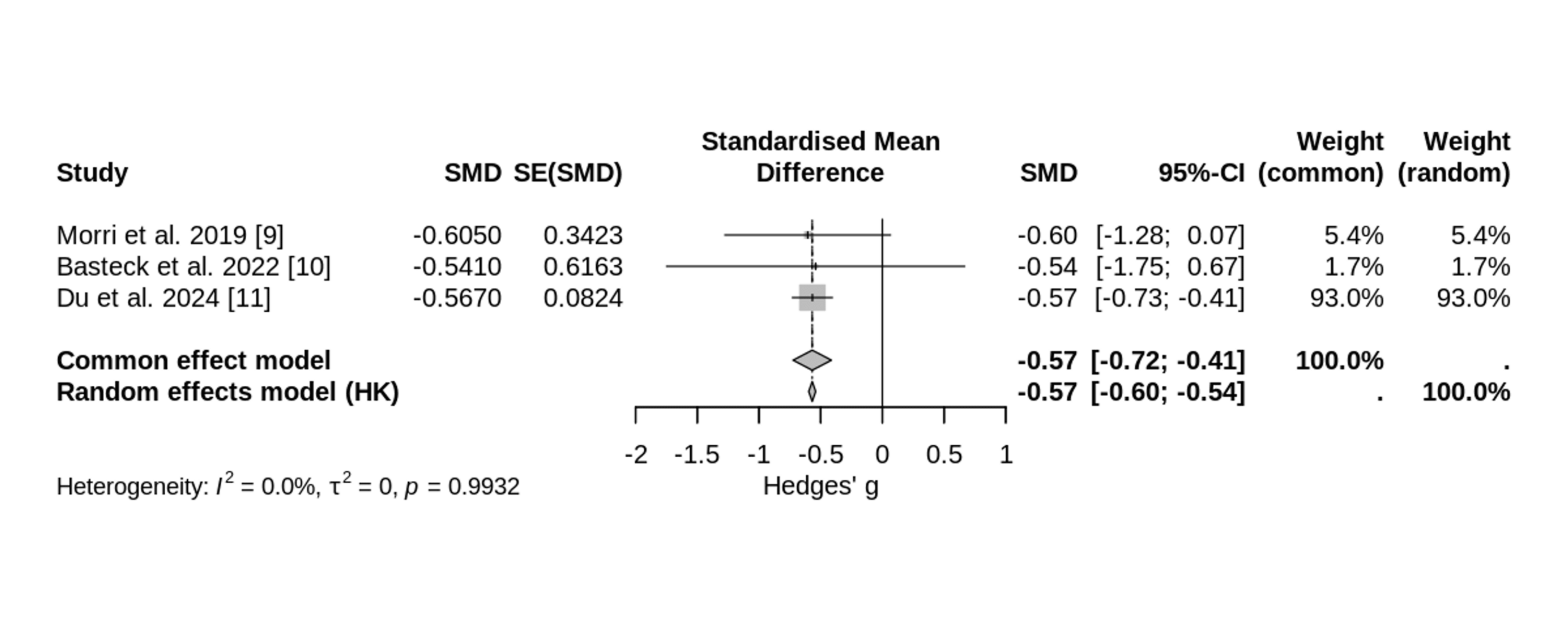
Forest Plot of Pooled Effect Size for Timed Up and Go (TUG) Test Following Exercise-Based Rehabilitation. Random-effects meta-analysis of TUG test data from three clinical trials (n = 576). The pooled Hedges’ g of –0.569 (95% CI: –0.597 to –0.540) indicates a significant benefit of exercise training compared to control. Individual study effect sizes with 95% confidence intervals are displayed alongside overall pooled estimate.

The funnel plot showed no evidence of bias (Figure 2). The meta-analysis of all TUG data yielded a pooled Hedges' g of -0.569 (95% CI (-0.597, -0.540)), favoring exercise over control (Figure 3). Sensitivity analyses confirmed robustness of findings (Table 6).

**Table 6:** Sensitivity Analysis of Pooled Effect Size for Timed Up and Go (TUG) Test by Excluding Individual Studies. Results of leave-one-out sensitivity analyses assessing the stability of the pooled effect size (Hedges’ g) for the TUG test. Each row shows the test statistics including pooled effect size, 95% confidence interval (CI), z-value, and p-value after excluding one study at a time. The findings demonstrate that the overall significant benefit of exercise-based rehabilitation remains robust despite the exclusion of any single study. The p-values < 0.001 were below the reporting threshold and are presented as such.

Excluded Study	Hedges' g	CI Lower	CI Upper	Z-value	p-value
Morri et al., 2019 [9]	-0.567	-0.727	-0.406	-6.937	< 0.001
Basteck et al., 2022 [10]	-0.569	-0.726	-0.412	-7.104	< 0.001
Du et al., 2024 [11]	-0.590	-1.176	-0.003	-1.971	0.049

Discussion

This meta-analysis provides novel and clinically relevant insights into the effects of exercise-based rehabilitation on gait function following endoprosthetic knee replacement for bone tumors. Notably, it is the first study to demonstrate a significant enhancement in functional mobility, measured via the TUG test, attributable to exercise interventions. The clinical relevance of this finding is underscored by prior research linking TUG performance to lower fall risk [12,13]. In contrast, kinematic parameters such as the GDI and GPS showed smaller, non-significant changes. This distinction highlights that while overall gait quality improvements may be subtle or variable, functional mobility as assessed by TUG appears responsive to targeted rehabilitation.

The raw difference in TUG across the three studies ranged from 0.40 to 1.70 seconds. The pooled effect size for TUG (Hedges’ g = -0.57) reflects a moderate and statistically significant benefit of exercise compared to control conditions, favoring rehabilitation protocols that begin early in the postoperative period. There was no meaningful heterogeneity among studies (I² = 0%; Q = 0.01; p = 0.993), indicating consistency in effect sizes, possibly due to the small number of studies, limited power, and unequal weights to detect heterogeneity.

Sensitivity analyses further confirmed the robustness of these findings, with the significant benefit persisting despite exclusion of individual studies. This strengthens confidence in the observed effect and suggests that exercise-based rehabilitation is an effective strategy to improve mobility and functional independence in this patient population.

Limitations of the study include the small number of included clinical trials (n = 3), which varied in sample size, study designs, and intervention protocols. The studies by Morri et al. (2019) and Basteck et al. (2022) were underpowered, limiting generalizability and their contribution to heterogeneity [9,10]. The clinical trial by Du et al. (2024) contributed the majority of participants and heavily influenced the meta-analytic results. These trials also lacked blinding in the intervention process [11]. Outcome measures beyond TUG, such as MSTS and TESS scores, demonstrated smaller and non-significant effects, possibly reflecting insensitivity to changes or differences in intervention targets. This was consistent with the functional outcomes described in a previous study [8].

Our findings also emphasize the importance of early initiation of exercise rehabilitation immediately post-surgery, which was associated with greater improvements in gait speed and postural control compared to delayed interventions starting one year postoperatively. This suggests a critical window for neuromuscular recovery and functional adaptation that rehabilitation protocols should target. Similarly, early rehabilitation has been shown to enhance gait in total knee replacement patients [14].

We summarize existing exercise-based rehabilitation protocols for endoprosthetic distal femur and proximal tibia reconstructions [9-11,15]. Furthermore, based on the kinematic deficits and muscle impairments inherent to each reconstruction site described in the Introduction, which collectively impair lower-limb stability and increase fall risk [16,17], we propose rehabilitation protocols that optimize functional recovery. The kinematics and muscle adaptation goals are summarized by site in Table 7. The primary rehabilitation targets in cases of proximal tibia reconstruction focus on protecting and gradually restoring the extensor mechanism, preserving the integrity of the medial gastrocnemius flap, strengthening ankle plantar flexors, and improving hip and ankle stability to compensate for muscle weaknesses [18]. In contrast, rehabilitation emphasis in femur cases is placed on promoting early knee range of motion within safe limits, enhancing quadriceps strength to support knee stability, and facilitating functional mobility through progressive weight-bearing and task-specific training [19-21]. Our proposed exercise principles are summarized in Table 8 by phases. This synthesis can guide practitioners in designing effective, evidence-based rehabilitation strategies tailored for this unique population.

**Table 7:** Kinematic and Muscle Adaptation Goals by Reconstruction Site. This table summarizes the key kinematic deficits and muscle adaptation targets for patients following proximal tibia and distal femur reconstructions. It highlights strategies to correct ankle dorsiflexion abnormalities, address muscle weaknesses, and optimize hip and knee extension mechanics to improve functional stability and gait.

Target	Tibia	Femur
Correct ankle dorsiflexion	Address plantarflexor weakness from gastrocnemius flap	Address co-contraction from quad inhibition
Hip/knee extension	Prevent stance collapse via quad strengthening	Ensure smooth flexion-extension transition

**Table 8:** Rehabilitation Phases and Focus Areas for Proximal Tibia and Distal Femur Reconstructions. This table outlines the sequential rehabilitation phases, specifying targeted therapeutic focuses for patients undergoing proximal tibia versus distal femur endoprosthetic reconstructions. It emphasizes phase-specific goals such as surgical protection, range of motion (ROM) control, muscle strengthening, and functional gait normalization tailored to the unique biomechanical demands of each reconstruction site.

Phase	Focus	Tibia	Femur
1	Protect surgery, reduce inflammation	Avoid >30° flexion, protect extensor mechanism	Earlier ROM possible, prevent stiffness
2	Gradual ROM, begin control	Emphasize safe extension	Promote flexion recovery
3	Functional strength, balance	Enhance knee extension, stabilize tibia	Strengthen quads, maintain full ROM
4	Normalize gait, advanced training	Restore full function, reduce compensations	Enable flexion-demanding tasks (e.g., stairs, sit-to-stand)

We present details of rehabilitation in different phases as follows (summarized in the Appendices).

Phase 1 (Immediate Postoperative; POD1-6 Weeks)

The initial rehabilitation phase prioritizes surgical site protection, inflammation reduction, and early muscle activation without compromising soft tissue healing. For tibia patients, knee flexion is restricted to less than 30 degrees with the brace locked in extension to safeguard the compromised extensor mechanism. Ankle pumps and isometric exercises targeting the gluteal and quadriceps muscles are introduced early to maintain muscle engagement and circulation. Weight-bearing is limited to toe-touch or partial, as tolerated, to reduce joint stress.

Femur patients benefit from earlier initiation of passive knee range of motion up to 60 degrees and gradual progression to partial weight-bearing, with a focus on patellar mobilization and isometric quadriceps and hamstring activation.

Incorporating functional task-oriented exercises such as "get up and sit down" (15 repetitions) and knee extensor strengthening in sitting using a theraband (60 repetitions over 5 minutes) promotes early activation of key muscle groups, particularly the quadriceps, while maintaining safety through controlled, low-load movements. Additionally, balance training activities such as side stepping, braiding exercises, and tandem walking, each performed for approximately 5 minutes, can be safely introduced during this phase to stimulate proprioception and improve neuromuscular control without risking joint instability. These functional and balance tasks complement traditional isometric strengthening and passive range of motion exercises by preparing patients for progressive weight-bearing and more dynamic activities in subsequent phases.

Phase 2 (Early Rehabilitation; Weeks 7-12)

This phase emphasizes controlled progression of the knee range of motion and initiation of muscle strengthening alongside proprioceptive training. Tibia patients cautiously increase active knee flexion from 30 to 60 degrees while maintaining protection of the extensor mechanism through isometric and concentric quadriceps strengthening below 90 degrees of flexion. Balance activities such as foam surface exercises and center shifting are introduced to enhance postural control. Weight-bearing progresses gradually based on patient tolerance and surgical stability.

For femur patients, the active range of motion extends up to 90 degrees, and strengthening includes concentric and eccentric exercises targeting the quadriceps and hip abductors. Proprioceptive training intensifies with the use of balance boards and initiation of gait training, transitioning from partial to full weight-bearing as tolerated.

Functional task-oriented exercises such as unilateral knee flexion to 90 degrees in standing (15 repetitions) and climbing on a platform or stairs (30 repetitions) help improve muscle strength and dynamic stability under progressively increased load. Walking activities, including backward walking, walking on a slope, and lateral crossing of the lower limbs (10 repetitions on each side, 5 minutes), enhance neuromuscular coordination and proprioception. Cardiovascular endurance and lower-limb coordination are further supported by stationary bicycle exercise or ergometry and treadmill walking (each for 10 minutes). Complementary balance training progresses with cross-over steps, shuttle walking, multiple changes in direction (each 5 minutes), and more challenging proprioceptive tasks such as foam activities, BAPS or tilt board exercises, and balance beam walking (each 10 minutes). Together, these interventions promote safe functional recovery, improving muscle strength, joint mobility, balance, and gait stability necessary for advancing to more demanding activities in later phases.

Phase 3 (Strength and Function; Months 3-6)

The focus shifts to dynamic strength development, normalization of proprioception, and functional gait restoration. Both tibia and femur patients engage in quadriceps strengthening within 120 degrees of knee flexion, stair-stepping exercises, and advanced balance training, including single-leg stance on unstable surfaces, eyes-closed tasks, and dual-task coordination exercises. Gait training emphasizes proper knee extension during heel strike and smooth terminal stance knee flexion. Weight-bearing is generally full, and patients begin incorporating more demanding functional activities to restore independence.

Phase 4 (Advanced Functional Training; Months 6-12)

In this final phase, rehabilitation targets the normalization of walking mechanics, restoration of activities of daily living, and readiness for return to work or low-impact sports. Tibia patients continue functional tasks requiring knee extension strength and control, such as sit-to-stand transitions, squatting, and stair descent, with balance training progressing to complex tasks involving cognitive dual-tasks. Femur patients participate in simulated occupational and recreational tasks emphasizing gait mechanics and neuromuscular control, while avoiding high-impact loading on the prosthesis to protect implant longevity.

In conclusion, this meta-analysis highlights the beneficial effects of exercise-based rehabilitation on functional mobility following endoprosthetic knee replacement, with the strongest evidence supporting early intervention to enhance gait speed and balance. Future research should focus on conducting large, well-designed randomized controlled trials with standardized outcome measures to confirm and expand upon these findings, ultimately improving rehabilitation care for patients with bone tumors.

## Conclusions

This review and meta-analysis provide evidence that structured exercise training significantly improves gait function and balance control in patients undergoing endoprosthetic knee replacement for bone tumors. The pooled results, including a moderate effect size for the TUG test (Hedges’ g = -0.569), support the clinical value of exercise interventions in enhancing postoperative mobility. Importantly, early initiation of rehabilitation, particularly within the immediate postoperative period, appears to yield greater functional benefits than delayed programs. These findings reinforce the need for early, targeted, and progressively challenging rehabilitation strategies to optimize gait outcomes and long-term quality of life in this patient population. Integration of such protocols into standard post-oncologic orthopedic care may substantially improve survivorship outcomes and functional independence.

## Appendices

Rehabilitation details by phases and sites

Phase 1: Immediate Postoperative (POD1-6 Weeks)

The general principles are to reduce swelling and pain, begin early isometric control, and protect surgical site integrity (Table 9).

**Table 9:** Phase 1: Immediate Postoperative (POD1–6 Weeks). ROM: range of motion, POD: post-operative day.

Component	Tibia	Femur
Brace	Locked in extension	May allow early flexion with limits
ROM	No flexion >30° until week 7	Passive ROM from POD2
Exercises	- Ankle pumps - Isometric glutes & quads - Patellar mobilization	Same as tibia, but start passive knee ROM by POD2
Weight-bearing	Toe-touch → partial if stable	Earlier transition to partial loading

Phase 2: Early Rehabilitation (Week 7 to Month 3)

The goals are to improve joint mobility safely, begin muscle activation and control, enhance proprioception with partial loading, protect tibial patients from developing extensor lag, and train femur patients to regain flexion symmetry (Table 10).

**Table 10:** Phase 2: Early Rehabilitation (Week 7 to Month 3). ROM: Range of motion, AROM: Active range of motion, WB: Weight-bearing.

ROM & Strength	Tibia	Femur
Knee ROM	Begin AROM 30° flexion	AROM 30°, more rapid progression
Quadriceps strengthening	Straight leg raise Isometric → active extension within brace	Leg raise + open chain up to 120° flexion
Balance and gait	Center shifting (20 reps × 3 sets); balance drills (Freeman board, foam mat); Wii Fit balance (once 50% WB allowed); ball toss dual tasking	Same as tibia, but can start earlier with increased ROM range

Phase 3: Strength and Function (Months 3-6)

The goals are to build dynamic strength, normalize proprioception and postural control, and initiate functional gait (Table 11). 

**Table 11:** Phase 3: Strength and Function (Months 3–6). ROM: range of motion.

Strengthening	Tibia	Femur
Quads	<120° ROM 10 sec × 20 reps × 3 sets	Same protocol
Stairs	Step up with the healthy leg (15 cm); step down with affected leg (10 cm)	Same
Balance progression	- Monopodalic on unstable surface - Eyes closed tasks - Dual-task throwing/catching - Obstacle clearance	Same, with focus on flexion in gait and squatting
Gait training	Partial → full weight-bearing Emphasize extension at heel strike	Emphasize terminal stance flexion range

Phase 4: Advanced Functional Training (Months 6-12)

The goals are to normalize walking mechanics, restore activities of daily living (ADLs) and recreational function, and achieve readiness for return to work or sport (Table 12).

**Table 12:** Phase 4: Advanced Functional Training (Months 6–12).

Advanced Training	Tibia	Femur
Gait retraining	Visual balance/gait feedback tools	Same
Functionality	Simulated work/home tasks; Carrying, stairs, balance with cognitive load	Emphasize squatting, stairs descent
Return to activity	Low-impact sport, light labor (if strength symmetrical)	Same
